# Examining the effectiveness of virtual, augmented, and mixed reality (VAMR) therapy for upper limb recovery and activities of daily living in stroke patients: a systematic review and meta-analysis

**DOI:** 10.1186/s12984-022-01071-x

**Published:** 2022-08-24

**Authors:** Sze Chit Leong, Yuk Ming Tang, Fong Mei Toh, Kenneth N. K. Fong

**Affiliations:** 1 Department of Industrial and Systems Engineering, The Hong Kong Polytechnic University, Hung Hom, Hong Kong, Hong Kong SAR; 2 Laboratory for Artificial Intelligence in Design, Hong Kong Science Park, New Territories, Hong Kong, Hong Kong SAR; 3 Department of Rehabilitation Sciences, The Hong Kong Polytechnic University, Hung Hom, Hong Kong, Hong Kong SAR

**Keywords:** Virtual reality, Augmented reality, Mixed reality, Stroke, Upper limb, Motor function

## Abstract

**Introduction:**

Virtual reality (VR), augmented reality (AR), and mixed reality (MR) are emerging technologies in the field of stroke rehabilitation that have the potential to overcome the limitations of conventional treatment. Enhancing upper limb (UL) function is critical in stroke impairments because the upper limb is involved in the majority of activities of daily living (ADL).

**Methods:**

This study reviewed the use of virtual, augmented and mixed reality (VAMR) methods for improving UL recovery and ADL, and compared the effectiveness of VAMR treatment to conventional rehabilitation therapy. The databases ScienceDirect, PubMed, IEEE Xplore, and Web of Science were examined, and 50 randomized control trials comparing VAMR treatment to standard therapy were determined. The random effect model and fixed effect model are applied based on heterogeneity.

**Results:**

The most often used outcomes of UL recovery and ADL in stroke rehabilitation were the Fugl-Meyer Assessment for Upper Extremities (FMA-UE), followed by the Box and Block Test (BBT), the Wolf Motor Function Test (WMFT), and the Functional Independence Measure (FIM). According to the meta-analysis, VR, AR, and MR all have a significant positive effect on improving FMA-UE for UL impairment (36 studies, MD = 3.91, 95 percent CI = 1.70–6.12, P = 0.0005) and FIM for ADL (10 studies, MD = 4.25, 95 percent CI = 1.47–7.03, P = 0.003), but not on BBT and WMFT for the UL function tests (16 studies, MD = 2.07, 95 percent CI = − 0.58–4.72, P = 0.13),

**Conclusions:**

VAMR therapy was superior to conventional treatment in UL impairment and daily function outcomes, but not UL function measures. Future studies might include further high-quality trials examining the effect of VR, AR, and MR on UL function measures, with an emphasis on subgroup meta-analysis by stroke type and recovery stage.

**Supplementary Information:**

The online version contains supplementary material available at 10.1186/s12984-022-01071-x.

## Background

Stroke is the world’s second greatest cause of death and the third-leading cause of disability in adults, and 80 million people worldwide suffer from the effect of a stroke [1]. Many stroke survivors suffer from a series of neurological sequelae, including physical, cognitive, and communication disorders. After a stroke, upper limb (UL) motor impairments are common, affecting approximately 80% of stroke survivors [2]. Full recovery of the hemiplegic upper limb function is difficult for most stroke survivors/patients, and this severely impairs their activities of daily living (ADL) and social involvement [3]. Enhancing the functional use of the upper limb after a stroke is important [4] because most tasks in everyday life involve the use of the upper limbs.

Despite the fact that conventional rehabilitation treatment has been shown to provide long-term benefits, patients are usually required to participate in very long-term treatments, and the results may vary depending on the experience of the individual therapists [5]. Patients, on the other hand, can lose motivation for treatment adherence since the treatment movements become tiresome and monotonous with time [6]. The emergence of innovative technologies, including virtual reality (VR), augmented reality (AR) and mixed reality (MR), has improved the conventional rehabilitation environment [7]. These new ways of treatment are valuable and provide substantial benefits not only to motivate patients to participate in long-term treatments but also to standardize the quality of treatment for stroke survivors [8, 9].

For VR therapy, a virtual environment resembling a human is constructed utilizing computer technology. Virtual reality is evolving tremendously, providing increasingly realistic virtual settings, which the user simply accepts while employing these therapies to induce recovery [10], while AR enables individuals to interact with virtual models with the use of a smart device such as a smartphone or tablet. The integration of the actual and virtual worlds enabled by augmented reality has the potential to enable humans to uncover abstract theories, phenomena, processes, and behaviors, as well as characteristics that are generally unavailable in a conventional clinical setting [11]. AR has been recognized as an emerging technology that, due to its ability to facilitate intense, repetitive and context-specific rehabilitation, can improve recovery after stroke [12]. For MR therapy, new digital technology in smart healthcare refers to a new type of environmental visualization created by fusing the actual world and the virtual digital world, in which physical entities and digital things can coexist and interact in real-time [13]. The MR system’s interactive media-based feedback provides an engaging medium for intuitively communicating performance and supporting the stroke survivor’s self-assessment [14].

For the early stages of recovery after stroke, virtual reality-based rehabilitation has received attention as a way to fill the gap between the real and ideal world due to its ability to provide high-intensity, repetitive and task-oriented training, as referenced by Kleim et al. [15]. In addition, Cho et al. [16] showed that the developed VR system can improve the motor control of stroke patients after VR proprioception feedback training. Virtual reality-based rehabilitation has shown similar progress to traditional physical therapy and occupational therapy [17]. Furthermore, this technology is an effective, feasible, and safe approach that simplifies rehabilitation compared to conventional rehabilitation, and creates a flexible and user-friendly interactive technique for demonstrating complicated and perplexing concepts [18]. With high-resolution medical consultation procedures and therapies, VR technology in medical applications can also help improve today’s healthcare systems [19]. Furthermore, a VAMR rehabilitation system provides a close collaborative system with high creativity, enhancing motor movements and minimizing the risk of patients feeling that the treatment is becoming tiresome and monotonous with time [20, 21]. The MR system’s evaluation and customizable feedback capabilities also allow clinicians to provide effective personalized training to patients [14].

As such, numerous systematic reviews have been undertaken to investigate the effectiveness of virtual reality on stroke rehabilitation. Wiley et al. [22] reviewed the use of VR technology that focused on the improvement of cognition and function, including global cognition, attention, memory, and language tests, however with a small number of studies covered, the meta-analysis results were highly affected by studies with a large population, causing lower accuracy. Another review conducted by Lee et al. [23], mainly investigated the effects of function in stroke patients. In their study, most of the cases included used game programs in VR intervention groups, which is less diverse. In addition to a review by Chen et al. [24], it analyzed the effects of balance control in stroke. Only nine studies were used, and most of the study groups had a relatively small sample size. Since it lacked external validity, the recorded results may not be relevant to a broader population. Moreno et al. [25] provided another overview of the literature on describing VR technology information for stroke rehabilitation. However, no quantitative analysis of the impacts was conducted, and the instruments and measures employed in the intervention were not been described. Therefore, we review the VAMR training that has effects on the recovery of upper limb function and ADL in order to generalize the findings. Our research is not only focused on the use of general virtual and augmented realities in rehabilitation treatment, but also includes the application of MR in order to investigate the impact of treatments using immersive technologies.

VR therapy is proven to be a worthwhile treatment for stroke patients, and our review aimed to address the following key research questions:

- RQ1: How virtual, augmented and mixed realities are used as interventions to improve hemiparetic UL function and ADL after stroke;
- RQ2: How does the effectiveness of VAMR therapies compare with conventional rehabilitation treatment for UL function after a stroke by meta-analysis.

This review has significant contributions: (1) not only in the review of VR and AR stroke rehabilitation, but also in investigating how MR can be used for rehabilitation; and (2) identification of the stroke outcome measurement scales used for the VAMR interventions. This study investigates VAMR on upper limb stroke rehabilitation, while fewer MR studies have been investigated in previous studies. More MR studies are included in the paper, examining the effects of the use of MR, identifying their significance and limitations, thus enhancing more future ideas for using MR for upper limb stroke rehabilitation. Furthermore, it is important to identify the most commonly used measures having high reliability for VAMR studies, so further research can focus on their advantages and limitations.

## Methods

### Data sources and search strategy

This study was conducted based on Preferred Reporting Items for Systematic Review and Meta-Analysis (PRISMA). From its inception to October 15, 2021, electronic database searches were conducted on ScienceDirect, PubMed, Web of Science and IEEE Xplore. We also manually searched the reference lists of related articles, and searched the databases using the following terms in Additional file 1: Table S1. Thus, using online screening software Mendeley Desktop to filter the titles and abstracts, and then view the titles and abstracts to assess whether the article meets our predetermined inclusion criteria.

### Studies flow of review

**Fig. 1:**
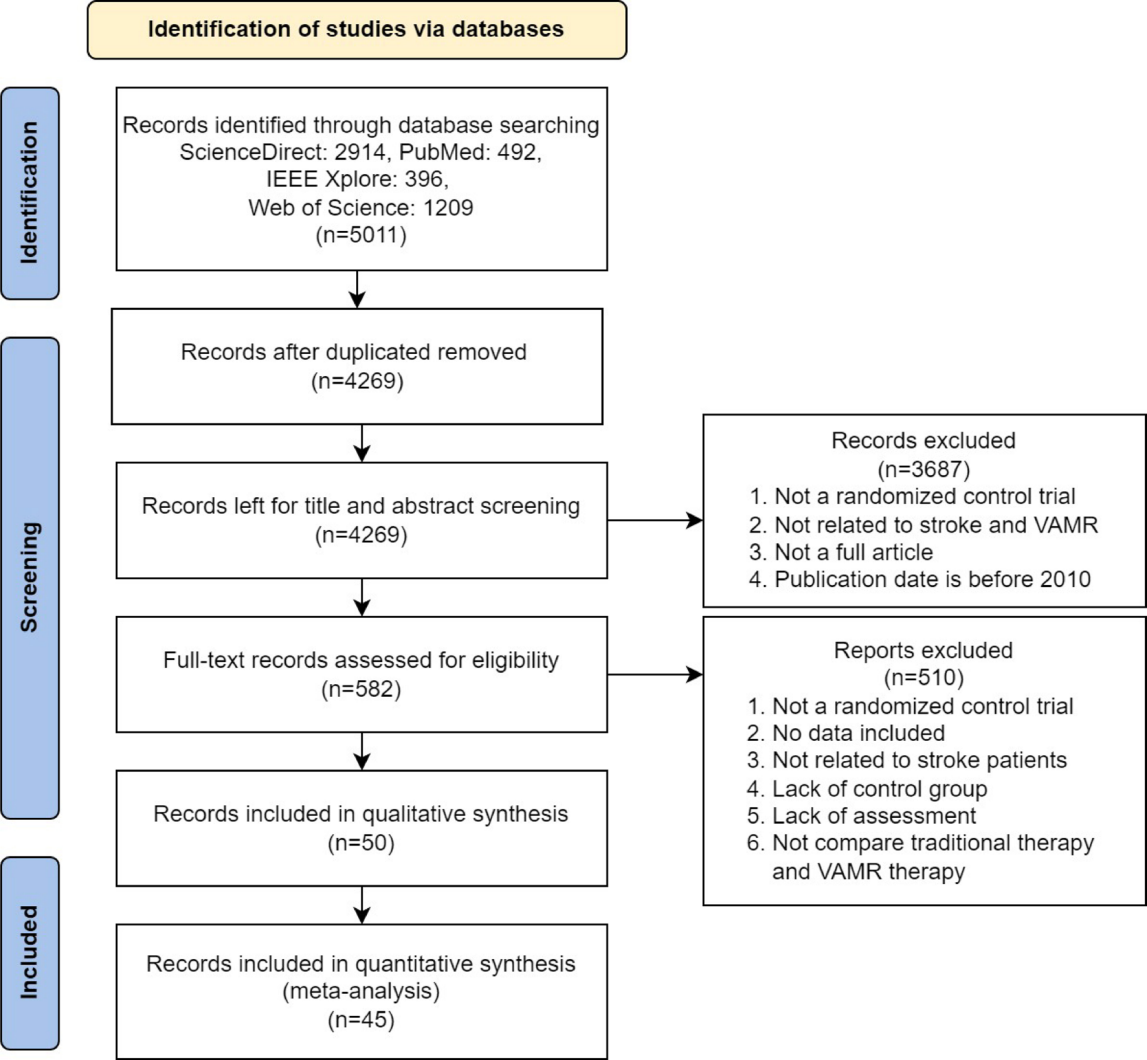
PRISMA Flowchart

Referring to the method description, 4 online databases were used to search for potentially relevant published articles, according to Fig. 1. The search strategy provided a total of 5,011 records. After removing duplicates, 4269 studies were further screened based on the titles and contents in the abstract. After manual full-text screening by two investigators, 50 randomized controlled trials met the inclusion criteria. All these studies compare VR, AR, or MR therapies with conventional treatment.

### Study selection

The eligibility of selected studies must meet the following inclusion criteria:

- *Participants* Eligible study participants were adults older than 18 years old, who had been diagnosed with a stroke. The study participants were not filtered according to the time after stroke, type of stroke, location of the lesion, or initial upper limb severity.
- *Study design* The inclusion criterion was a randomized controlled trial (RCTs), which divided patients into an experimental group receiving either VAMR treatment or a control group receiving conventional treatment.
- *Outcome Measures* Any method of measuring the physical, mental and social functions of an individual.
- *Language *Articles are published in English.

For the exclusion criteria, book chapters, conference papers and abstracts are excluded. Duplicates and qualitative studies are also excluded. Furthermore, trials comparing two VR groups without control conditions are excluded.

### Quantitative analysis

The average post-treatment score, standard deviation, and group size of each comparable trial were entered into RevMan software version 5.4. The summary results were evaluated by calculating the mean difference (MD) with a 95% confidence interval (CI). Since the studies within the same meta-analysis used the same assessment tool with the same unit of measurement, the mean difference (MD) was used as a summary statistic in the meta-analysis when the outcome measurements in all studies were made on the same scale and a fixed-effect model was used. When there was a high degree of heterogeneity between trials (I^2^ > 50%), the random-effect model was used to pool trial findings for outcomes [26]. To illustrate the pooled effect, forest plot graphics were generated. All tests were two-sided, and we regard a P-value < 0.05 to be statistically significant. Furthermore, RevMan 5.4 was used to analyze the publication bias in this study.

For the quality assessments, the Physiotherapy Evidence Database (PEDro) scale was used to assess the quality of each study [27]. On the basis of the following classification, studies were ranked as excellent to poor: A score of 9–10 was considered excellent; a score of 6–8 was considered good; a score of was 4–5 considered fair; and a score of less than 4 considered poor; the study involved articles with a score of greater than or equal to 4 [27].

## Results

To address the key research questions, the results were divided into three sections intended to address the key RQs; including how VAMR therapies improved UL recovery in stroke rehabilitation, what the stroke outcome measure scales used, and the effectiveness of VAMR therapies. A table containing the clinical information and outcome measures of studies on VAMR and conventional treatment was used to summarize the findings, followed by a discussion of VAMR therapies. The second research question on investigating the most used outcome measures scale for UL functions in stroke is discussed next. Lastly, four outcome measures including FMA-UE, BBT, WMFT, and FIM were selected to perform the meta-analysis and compare the effectiveness between VAMR and conventional treatment in UL impairment, function, and ADL measures.

### The treatment used in studies

To investigate how VAMR improves UL function in stroke rehabilitation, Table 1 summarizes the clinical information and outcome measures of studies on VAMR and conventional treatment. The 50 studies in our review included a pooled sample of 2271 participants of 3 [28] to 263 [29] participants in each group. All types of strokes were included in this study. The average age of the sample was 45–75.59 years. Thirty-eight studies only reported the results before and after the intervention, and the remaining studies also included follow-up measurements 1 month after the intervention.

**Table 1:** Clinical information and outcome measures of studies on VAMR and traditional therapy

Author	No. of participants	Mean age (SD)	Recovery stage	Experimental Intervention	Control Intervention	Outcome Measures
Ahmad et al. 2019 [30]	EG: 18	57	Chronic	1.5 h of standard physiotherapy exercise with 30 min VR training (once a week for 8 weeks)	2 h of standard physiotherapy exercise (once a week for 8 weeks)	FMA-UE/WMFT/IMI/IADL/SIS
	CG: 18	62.94				
Aşkın et al. 2018 [31]	EG: 18	53.27 ± 11.19	Chronic	20 sessions of physical therapy + 20 sessions of Kinect-based VR training	20 sessions of physical therapy	FMA-UE/BBT/BRS/MAS/MI/AROM
	CG: 20	56.55 ± 9.85				
Assis et al. 2016 [28]	EG: 3	50.5	Chronic	EG sensory-motor training in using NeuroR system (4 weeks)	Having a relaxation session, instructed by the physiotherapist (4 weeks)	FMA-UE
	CG: 3	59.5				
Bergmann et al. 2017 [32]	EG: 10	62 ± 11	Subacute	VR-augmented robot-assisted gait training (RAGT) 12 sessions (4 weeks, 3 sessions per week)	Standard RAGT 12 sessions (4 weeks, 3 sessions per week)	IMI/FAC/MRC
	CG: 10	65 ± 8				
Brunner et al. 2017 [33]	EG: 62	62	Subacute	60 min VR training (30 days, 4–5 sessions per week)	60 min standard conventional therapy program (30 days, 4–5 sessions per week)	ARAT/BBT/FIM/PGIC
	CG: 58	62				
Byl et al.2013 [34]	EG: 10	65.2 ± 5.4	Chronic	Perform repetitive movements while playing task-specific games (Twice a week for 6 weeks)	Repetitive task exercises involve stretching, and grasping (Twice a week for 6 weeks)	FMA-UE/WMFT/BBT
	CG: 5	54.2 ± 20.5				
Calabrò et al. 2017 [35]	EG: 12	60 ± 4	Chronic	Lokomat with VR (RAGT + VR)	Lokomat without VR (RAGT-VR)	RMI/POMA/MAS/HRS/VAS
	CG: 12	63 ± 6				
Cameirão et al. 2011 [17]	EG: 10	56.8	Acute	Perform the Spheroids tasks (Hitting, Grasping, and Placing) (Once a week for 3 weeks)	Perform pure extended occupational therapy (Once a week for 3 weeks)	FMA-UE/MBI/MRC/MI
	CG: 9	52.9				
Chen et al. 2015 [36]	EG: 8	58.2 ± 12.1	Chronic	30 min play bowling and ladder climbing games by XaviX®Port system (3 sessions per week for 8 weeks)	30 min in use of Curamotion exerciser and the climbing board and bar (3 sessions per week for 8 weeks)	FMA-UE/FIM/BBT/ROM
	CG: 8	48.5 ± 16.4				
Cho et al. 2012 [37]	EG: 15	64.0 ± 7.1	N/A	VR training (60 min sessions, 5 times a week, for 4 weeks)	Traditional rehabilitation (30 min sessions, 3 times a week, for 4 weeks)	WMFT/MVPT
	CG: 14	63.7 ± 8.8				
Choi et al. 2016 [38]	EG: 12	61.0 ± 15.2	N/A	30 min occupational therapy + 30 min MoU-Rehab (5 days per week for 2 weeks, 1 h per day)	1 h occupational therapy (5 days per week for 2 weeks, 1 h per day)	FMA-UE/B-stage/MMT/MBI/EQ-5D/BDI
	CG: 12	72.1 ± 9.9				
Duff et al. 2013 [14]	EG: 11	69.27 ± 7.85	Chronic	Adaptive mixed reality rehabilitation (AMRR) system (3 times a week for 4 weeks)	1-h upper-extremity therapy (3 times a week for 4 weeks)	FMA-UE/WMFT/MAL/SIS
	CG: 10	67.7 ± 7.85				
El-Kafy et al. 2021 [39]	EG: 18	53.32 ± 5.13	Chronic	2 h muscle exercises, ADL tasks, and VR training program using Armeo Spring (3 times a week for 3 months)	2 h conventional functional training program (3 times a week for 3 months)	ARAT/WMFT/HGS
	CG: 19	54.46 ± 4.27				
Faria et al. 2018 [40]	EG: 12	57.1 ± 11.0	Chronic	Underwent training with the Reh@Task (for 1 month)	Conventional occupational therapy (for 1 month)	MoCA
	CG: 12	68.9 ± 9.8				
Ho et al. 2019 [41]	EG: 100	67.97 ± 11.38	Acute	40 min conventional therapy + 20 min VR program (7 times for 1 week)	1 h conventional therapy only (7 times for 1 week)	mRS/NIHSS
	CG: 100	67.68 ± 11.13				
Hung et al. 2019 [42]	EG: 17	56.58	Chronic	30 min Kinect2scratch with 3–4 games per training session (2/3 sessions per week, total 24 sessions)	30 min conventional therapy (2/3 sessions per week, total of 24 sessions)	FMA-UE/WMFT/MAL
	CG: 16	61.38				
Ikbali Afsar et al. 2018 [43]	EG: 19	69.42 ± 8.55	Subacute	VR training using Xbox Kinect + conventional therapy (5 days per week, for 4 weeks)	Conventional rehabilitation program (5 days per week, for 4 weeks)	FMA-UE/BBT/FIM
	CG: 16	63.44 ± 15.73				
In et al. 2012 [44]	EG: 11	63.45 ± 11.78	Chronic	30 min VR reflection therapy (5 days a week for 4 weeks)	30 min conventional therapy (5 days a week for 4 weeks)	FMA-UE/BBT/MAS/MFT
	CG: 8	64.50 ± 11.69				
Johnson et al. 2020 [45]	EG: 28	64.7 ± 13.9	Chronic	45 min Jintronix Rehabilitation System (2 times a week for 8 weeks)	Usual care (2 times a week for 8 weeks)	FMA-UE/ARAT/BBT/MAS
	CG: 30	59.3 ± 15.6				
Kalron et al. 2016 [46]	EG: 15	47.3 ± 9.6	N/A	30 min Computer Assisted Rehabilitation Environment (CAREN) Integrated Reality System with D-flow software (6 weeks, 2 sessions per week)	10 min stretching exercises + 20 min intervention (6 weeks, 2 sessions per week)	FRT/BBT/FSST
	CG: 15	43.9 ± 10.6				
Kim et al. 2018 [47]	EG: 11	VR: 54.7 ± 17.3	Subacute	30 min occupational therapy + 30 min daily VR (5 days per week for 10 weekdays)	30 min occupational therapy (5 days per week for 10 weekdays)	FMA-UE/B-stage/BBT/K-MBI
	CG: 8	CG:53.5 ± 16.0				
Kiper et al. 2011 [48]	EG: 40	All: 64 ± 16.4	Chronic	1 h traditional neuromotor rehabilitation treatment + 1 h reinforced feedback in virtual environment therapy (5 days a week for 4 weeks)	2 h traditional neuromotor rehabilitation treatment (5 days a week for 4 weeks)	FMA-UE/FIM/ASS
	CG: 40					
Kiper et al. 2014 [49]	EG: 23	63.1 ± 9.5	Chronic	1 h VR treatment + 1 h conventional treatment (5 days per week for 4 weeks)	2 h of conventional training (5 days per week for 4 weeks)	FMA-UE/FIM
	CG: 21	65.5 ± 14.2				
Kottink et al. 2014 [50]	EG: 8	N/A	Chronic	30 min VR rehabilitation game (Once per week for 6 weeks)	30 min conventional training (Once per week for 6 weeks)	FMA-UE
	CG: 10					
Lee et al. 2014 [51]	EG: 12	58.33 ± 10.17	N/A	30 min virtual reality reflection equipment (Asymmetric training on hand) + 1 h standard rehabilitation training (4 weeks)	30 min symmetric training on hand + 1 h standard rehabilitation training (4 weeks)	FMA-UE/BBT/MAS/ROM
	CG: 12	65.42 ± 9.77				
Lee et al. 2016 [52]	EG: 13	66.46 ± 7.26	Chronic	30 min VR rehabilitation program (3 times per week for 8 weeks)	30 min conventional training sessions (3 times per week for 8 weeks)	FMA-UE/MFT/BBT/MBI/ SF-12
	CG: 13	69.92 ± 7.18				
Lee et al. 2018 [53]	EG: 15	VR: 61.80 ± 6.80	Subacute	VR canoe paddling training (30 min each day, 3 times per week, for 5 weeks)	Conventional rehabilitation program (30 min each day, 3 times per week, for 5 weeks)	mFRT// MFT
	CG: 15	CG: 61.33 ± 8.44				
Levin et al. 2012 [54]	EG: 6	58.1 ± 14.6	Chronic	VR therapy (goal-directed reaching tasks) (4 clinical evaluations + 9 intervention sessions)	Occupational therapy (4 clinical evaluations + 9 intervention sessions)	FMA-UE/BBT/WMFT/CSI
	CG: 6	59.8 ± 15.1				
Lin et al. 2018 [55]	EG: 5	45.0 ± 11.2	N/A	35 min VR game with a motion tracking device (12 sessions, 3 sessions per week)	35 min traditional rehabilitation (12 sessions, 3 sessions per week)	FMA-UE
	CG: 5	52.2 ± 7.7				
Lin et al. 2020 [56]	EG: 38	VR: 64.5 ± 13.5	Acute	3–6 days early rehabilitation + 5 days VR Training	3–6 days early rehabilitation	MBI/PASS/HADS
	CG: 107	CG: 66.9 ± 13.3				
ÖGÜN et al. 2019 [57]	EG: 33	61.48 ± 10.92	Chronic	60 min of the upper extremity immersive VR rehabilitation program	45 min of conventional therapy and 15 min of a sham VR program	ARAT/FMA-UE/FIM/PASS
	CG: 32	59.75 ± 8.07				
Oh et al. 2019 [58]	EG: 17	57.4 ± 12.2	Chronic	VR combined real instrument training (6 weeks)	Conventional occupational therapy (6 weeks)	MMT/mAS/FMA-UE/K-MMSE/K-MoCA/BBT
	CG: 14	52.6 ± 10.7				
Park et al. 2016 [59]	EG: 15	61.6 ± 5.34	Chronic	30 min game-based VR movement therapy using the Wii (5 days a week for 4 weeks)	30 min conventional therapy (5 days a week for 4 weeks)	FMA-UE/BBT/MAL-QOM
	CG: 15	62.0 ± 4.29				
Park et al. 2019 [60]	EG: 12	53.5 ± 13	Subacute	30 min using Smart Board (20 sessions, 5 days per week, 4 weeks)	30 min using conventional occupational therapy (20 sessions, 5 days per week, 4 weeks)	FMA-UE/WMFT/AROM/MBI
	CG: 13	51.5 ± 16.7				
Pedreira da Fonseca, 2017 [61]	EG: 15	53.8 ± 6.3	Chronic	Use Nintendo Wii for VR therapy in 45 min + 15 min conventional therapy	1 h conventional therapy	DGI
	CG: 15	50.9 ± 10.9				
Piron et al. 2010 [62]	EG: 27	59 ± 8	N/A	Perform motor tasks with real objects by using 3D magnetic receiver to record movement (5 days per week for 4 weeks)	Perform specific exercises for the arm (5 days per week for 4 weeks)	FMA-UE/FIM
	CG: 23	62 ± 10				
Prange et al. 2015 [63]	EG: 35	60.3 ± 9.7	Subacute	30 min arm support training by using ArmeoBoom (Once per week for 6 weeks)	30 min standardized sets of arm exercises + OT (Once per week for 6 weeks)	FMA-UE/SULCS
	CG: 33	58 ± 11.4				
Saposnik et al. 2010 [64]	EG: 11	55	Chronic	Use the Nintendo Wii game console to play “Wii Sports” (20 h in 2 weeks)	Leisure activities, including cards, bingo and building blocks (20 h in 2 weeks)	WMFT/BBT/SIS
	CG: 113	67				
Saposnik et al. 2016 [65]	EG: 71	62 ± 13	Acute	Non-immersive virtual reality using the Nintendo Wii (2 weeks)	Simple recreational activities (2 weeks)	WMFT/BBT/MBI/FIM/SIS
	CG: 70	62 ± 12				
Shin et al. 2013 [66]	EG: 9	46.6 ± 5.8	N/A	RehabMaster™	Conventional occupational therapy	FMA-UE/MBI
	CG: 7	52 ± 11.9				
Shin et al. 2014 [67]	EG: 9	52.0 ± 11.9	N/A	Conventional occupational therapy + 20 min of RehabMaster training (10 sessions in 2 weeks)	Conventional occupational therapy (10 sessions in 2 weeks)	FMA-UE/MBI
	CG: 7	46.6 ± 55.8				
Shin et al. 2015 [68]	EG:16	53.37 ± 11.8	Chronic	30 min conventional therapy + 30 min of game-based VR rehabilitation with the RehabMasterTM system (5 days a week for 4 weeks)	30 min of conventional therapy + additional 30 min therapy (5 days a week for 4 weeks)	FMA-UE/HDRS
	CG: 16	54.67 ± 13.4				
Shin et al. 2016 [69]	EG: 24	57.2 ± 10.3	N/A	Smart glove intervention (4 weeks with 20 sessions for 30 min per day)	Conventional therapy (4 weeks with 20 sessions for 30 min per day)	FMA-UE/JTT
	CG: 22	59.8 ± 13				
Sin et al. 2013 [70]	EG: 18	71.78 ± 9.42	Chronic	Xbox Kinect with 30 min + conventional OT for 30 min (3 times a week for 6 weeks)	1 h conventional occupational therapy (3 times a week for 6 weeks)	FMA-UE/BBT/AROM
	CG: 17	75.59 ± 5.55				
Subramanian et al. 2013 [71]	EG: 16	62 ± 9.7	Chronic	3D virtual environment (CAREN system) simulated a supermarket scene (3 times a week for 4 weeks)	Point to the target in the physical environment (3 times a week for 4 weeks)	FMA-UE/WMFT/MAL-AS
	CG: 16	60 ± 11				
Thielbar et al. 2020 [72]	EG: 9	59.7 ± 10.5	Chronic	Virtual Environment for Rehabilitative Gaming Exercises system (3 evaluation sessions)	Conventional therapy (3 evaluation sessions)	FMA-UE
	CG: 9	59.8 ± 4.8				
Tramontano et al. 2018 [73]	EG: 13	63.1 ± 8.5	Subacute	20 min vestibular rehabilitation (12 sessions, 3 times per week for 4 weeks)	20 min conventional rehabilitation training (12 sessions, 3 times per week for 4 weeks)	MBT/FAC/BBS/RMI/T-total/T-balance
	CG: 12	65.1 ± 15.5				
Turolla et al. 2013 [29]	EG: 263	60.2 ± 14.3	N/A	1 h of upper limb conventional therapy and 1 h of VR therapy (4 weeks)	2 h conventional treatment (4 weeks)	FMA-UE/FIM
	CG: 113	65.4 ± 12.5				
Viana et al. 2014 [74]	EG: 10	56.0 ± 10.2	Subacute	1 h VR exercises for the UL + 13 min transcranial direct current stimulation (primary motor cortex) (5 weeks)	1 h transcranial direct current stimulation (primary motor cortex) (5 weeks)	FMA-UE/WMFT/MAS/SSQOL
	CG: 10	55.0 ± 12.2				
Yin et al. 2014 [75]	EG: 11	62	N/A	Nine 30 min upper extremity VR therapy + 30 min conventional therapy (5 weekdays for 2 weeks)	1 h conventional therapy (5 weekdays for 2 weeks)	FMA-UE/ARAT/MAL/FIM
	CG: 12	56				

ARAT, Action Research Arm Test; ASS, Ashworth scale score; BBT, Box and Block Test; BDI, Beck Depression Inventory; B-stage, Brunnstrom stage; DGI, Dynamic Gait Index; EQ-5D, EuroQol-5 Dimension; FAC, Functional Ambulation Categories; FIM, Functional Independence Measure; FMA-UE, Fugl-Meyer Upper Extremity; FRT, Functional Reach Test; FSST, Four Square Step Test; HADS, Hospital Anxiety and Depression Scale; HDRS, Hamilton Depression Rating Scale; IADL, The Lawton Instrumental Activities of Daily Living; IMI, Intrinsic motivation inventory; MMSE, Mini-Mental State Examination; MAL, Motor Activity Log; MAS, Motor Assessment Scale; MBI, Modified Barthel Index; MI, Motricity Index; MMT, Manual muscle testing; MoCA, Montreal Cognitive Assessment; MRC, Muscle Power Assessment; mRS, Modified Rankin Handicap Scale; MVPT, Motor-free Visual Perception Test; NIHSS, National Institutes of Health Stroke Scale; PASS, Postural Assessment Scale for Stroke; PGIC, Paramedic Global Impression of Change; POMA, Performance Oriented Mobility Assessment; RMI, Rivermead Mobility Index; ROM, Range of Motion; SF-12, 12-item Short Form Health Survey; SIS, Stroke Impact Scale; SSQOL, Stroke Specific Quality of Life Scale; SULCS, Stroke Upper Limb Capacity Scale; VAS, Visual analogue scale; WMFT, Wolf Motor Function Test

#### Characteristics of VAMR therapy

The experimental group uses many VAMR techniques and interventions, including canoe paddling training, standard physical therapy exercises with VR training, Reh@Task, MoU-Rehab, Smart Glove, Smart Board, Reh@City and Lokomat and VR, and CAREN integrates reality systems to simulate daily activities and daily tasks. In all trials, the intervention time was 1 [41] to 8 weeks [30, 36, 45, 76], the operation frequency was 1–7 times a week, and the duration was around 30–120 min.

An article stated that as participants’ abilities and entertainment levels improve, the difficulty of controlling interventions will gradually develop over time [77].

##### Virtual reality

The studies using VR can be divided into two main types: readily available commercial games, and a VR system designed for upper extremity rehabilitation.

For readily available commercial games, there are numerous types of readily available commercially released games in the market and the most common brands are Nintendo Wii and Xbox Kinect. Saposnik et al. [65], reported that they used Wii Sports and Game Party 3 games as their therapy method. Participants were able to select various tasks within certain games depending on their skills and interests as they progressed through the intervention, intending to improve endurance, range of movement, stamina, and coordination of the injured arm. The recreational activity was created as a standard active control with equal difficulty and sophistication to mimic the VR Wii group’s abilities while also encouraging motivation.

In another research study by Askin et al. [31], Xbox Kinect was used for rehabilitation, using an infrared camera to capture the body activity of players in 3D space for interaction within game events. The user’s body serves as a game controller in 3D space, allowing players with minimal motor abilities to engage in the game. The games “Good View Hunting” and “Hong Kong Chef” requires patients to move their hands to pick or remove objects to achieve a high score, and the patients actively practiced bilateral shoulder abduction and adduction, as well as active elbow flexion and extension motions.

For the VR system designed for upper extremity rehabilitation, there are numerous models of suitable VR systems nowadays, and most of them have similar components: Sensor, camera, monitor and VR programs or software. An example is the Lin et al. trial [56], in which therapeutic community counselling was performed in a private room in the neurological treatment ward using a portable VR interface and a Microsoft Corporation Kinect sensor. Numerous studies used customized VR games/systems for upper limb stroke rehabilitation. In Kiper’s study [49], the participant was seated next to the wall screen gripping a sensorized real object (e.g., ball, disk, or glass) with the paretic hand during the virtual reality diagnosis; in case of extreme inability in grasping, the sensor was attached to a glove worn by the user. The sensor system detects practical motions such as forearm pronation/supination, wrist flexion/extension, radial-ulnar deviation, and finger flexion/extension by tracking the motion and stance of the wearer’s distal limb [63]. With a tailored system, the VR system in Kiper’s study [48] provided high flexibility in rehabilitation for stroke patients according to their recovery progress.

##### Augmented reality

In Mousavi et al. [21] study, which used an AR-based version of the Fruit Ninja game, the game required subjects to perform quick arm movements that included visual and somatosensory inputs; however, in the AR-based version, respondents stared directly at their hand as it moved to control gameplay. In addition, an AR method may allow patients to safely perform real-life functional skills, as well as provide standardization and consistency across multiple trials, which are challenging to achieve in traditional therapy with real items.

##### Mixed reality

In the study by Duff et al. [14], an adaptive mixed reality rehabilitation (AMRR) system employed an interactive framework to teach motor components linked to task completion and movement quality. AMRR combines smart object-based repeated task training with real-time motion capture and analysis to extract kinematic measures that may be used to quantify arm motor performance and give a systematic assessment of common upper-extremity deficits. The kinematic data is also used to provide a participant with real-time and summary audio-visual feedback for self-assessment of the movement. The interactions are engaging in order to encourage task completion and enhance generalized learning of task-related motor aspects.

#### Characteristics of conventional rehabilitation treatment

Conventional therapy refers to the routine stroke care and treatment that stroke patients get as part of their rehabilitation. Control group intervention included conventional occupational therapy or physical therapy exercise and simple recreational activities. Physical therapy helped to enhance coordination and body control, while occupational therapy was used to improve activities of daily living skills (ADL). In these studies, the frequency of the control interventions was identical to those of the intervention groups.

### Meta-analysis of post-therapy

**Fig. 2:**
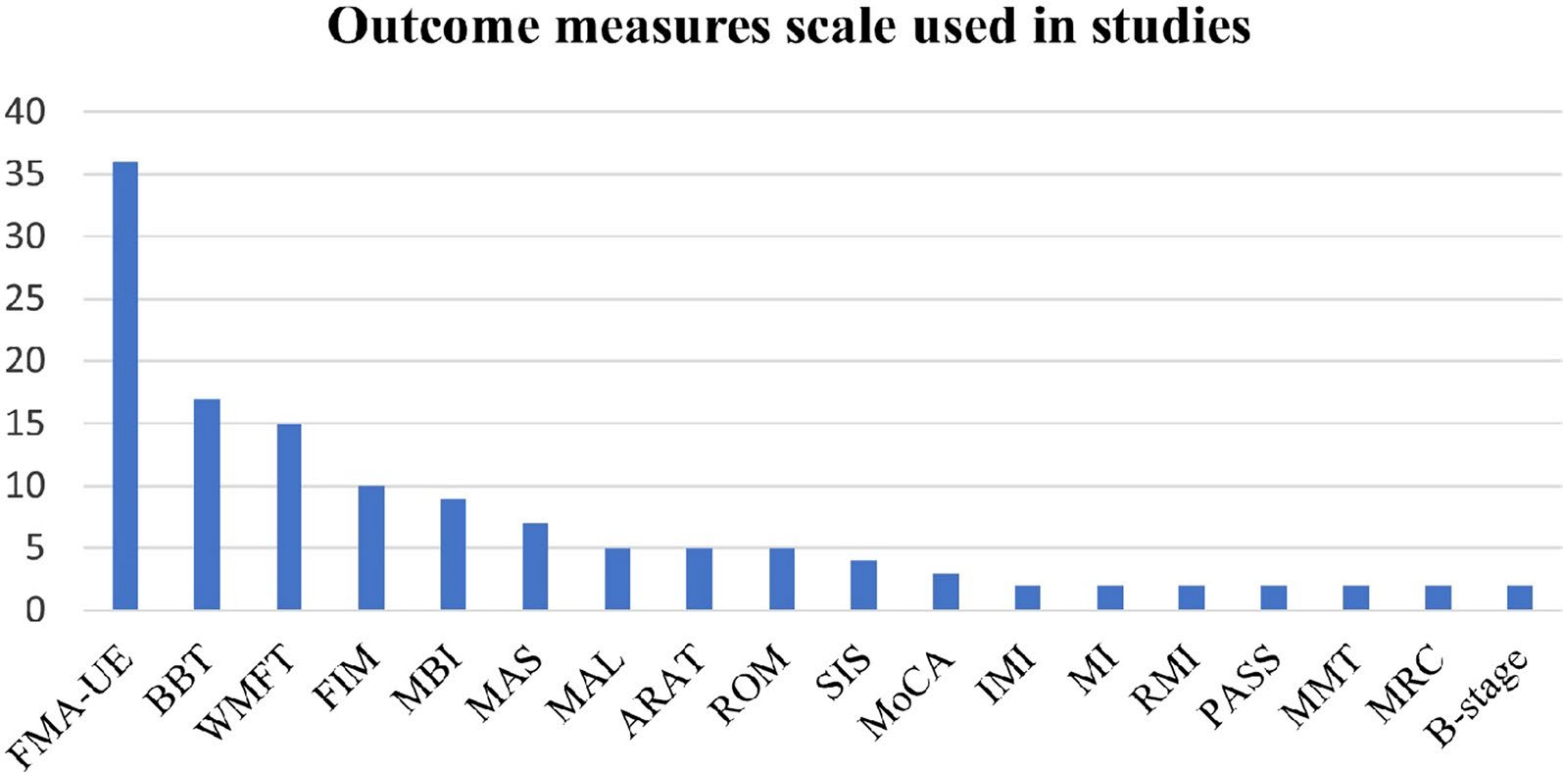
Outcome measures included in studies

To compare the effects of VAMR therapy with conventional therapy, it was analyzed by meta-analysis, by investigating the effectiveness of the intervention in improving the scores of the FMA-UE, BBT, WMFT and FIM, which are the most commonly used outcome measures of UL impairment, function, and ADL in the studies, refer to Fig. 2. The four outcomes are proven with high reliability and validity [37], and a meta-analysis used these outcome measures to compare VAMR and conventional therapies.

According to Table 2, the mean PEDro scale score for the studies included in this analysis was 6.28, ranging from 4 to 9, thus indicating that they were of high quality. 2 studies scored 4, 12 studies scored 5, 15 studies scored 6, 12 studies scored 7, 7 studies scored 8, and 2 studies scored 9.

**Table 2:** PEDro Scale risk of bias ratings for the included studies

Studies	Eligibility criteria	1	2	3	4	5	6	7	8	9	10	PEDro score
Ahmad et al. 2019 [24]	Y	1	0	1	0	0	1	1	1	1	1	7
Aşkın et al. 2018 [25]	Y	1	0	1	0	0	1	1	0	1	1	6
Assis et al. 2016 [26]	Y	1	0	1	0	0	0	1	1	1	1	6
Bergmann et al. 2017 [27]	Y	1	0	1	0	0	1	1	0	1	1	6
Brunner et al. 2017 [28]	Y	1	1	1	0	0	1	1	0	1	1	7
Byl et al.2013 [29]	N	1	0	1	0	0	1	0	0	1	1	5
Calabrò et al. 2017 [30]	Y	1	1	1	0	1	1	1	1	1	1	9
Cameirão et al. 2011 [14]	Y	1	0	0	0	0	1	0	0	1	1	4
Chen et al. 2015 [31]	Y	1	0	1	0	0	0	1	0	1	1	5
Cho et al. 2012 [32]	Y	1	0	1	0	0	0	1	0	1	1	5
Choi et al. 2016 [33]	Y	1	1	1	0	0	1	1	1	1	1	8
Duff et al. 2013 [34]	Y	1	0	1	0	0	1	0	0	1	1	5
El-Kafy et al. 2021 [35]	Y	1	0	1	0	0	1	1	0	1	1	6
Faria et al. 2018 [36]	Y	1	1	1	0	0	1	1	0	1	1	7
Ho et al. 2019 [37]	Y	1	1	1	0	0	0	0	0	1	1	5
Hung et al. 2019 [38]	Y	1	1	1	0	0	1	1	1	1	1	8
Ikbali Afsar et al. 2018 [39]	Y	1	1	1	0	0	1	0	0	1	1	6
In et al. 2012 [40]	Y	1	0	1	0	0	0	0	0	1	1	4
Johnson et al. 2020 [41]	Y	1	1	1	0	0	1	1	1	1	1	8
Kalron et al. 2016 [42]	N	1	1	1	0	0	1	1	0	1	1	7
Kim et al. 2018 [43]	N	1	1	1	1	0	1	0	1	1	1	8
Kiper et al. 2011 [44]	Y	1	0	1	0	0	0	1	0	1	1	5
Kiper et al. 2014 [45]	Y	1	0	1	0	0	0	1	0	1	1	5
Kottink et al. 2014 [46]	Y	1	0	1	0	0	1	1	0	1	1	6
Lee et al. 2014 [47]	Y	1	0	1	0	0	1	0	0	1	1	5
Lee et al. 2016 [48]	Y	1	1	1	0	0	1	1	1	1	1	8
Lee et al. 2018 [49]	Y	1	0	1	0	0	1	1	0	1	1	6
Levin et al. 2012 [50]	Y	1	0	1	0	0	1	1	0	1	1	6
Lin et al. 2018 [51]	Y	1	0	1	0	0	1	1	1	1	1	7
Lin et al. 2020 [52]	Y	1	1	1	1	0	0	1	1	1	1	8
ÖGÜN et al. 2019 [53]	Y	1	1	1	0	0	0	1	0	1	1	6
Oh et al. 2019 [54]	Y	1	1	1	0	0	1	1	0	1	1	7
Park et al. 2016 [55]	Y	1	0	1	0	0	1	1	0	1	1	6
Park et al. 2019 [56]	Y	1	1	1	0	0	1	1	0	1	1	7
Pedreira da Fonseca, 2017 [57]	Y	1	1	1	0	0	1	0	1	1	1	7
Piron et al. 2010 [58]	Y	1	1	1	0	0	1	1	1	1	1	8
Prange et al. 2015 [59]	Y	1	1	1	0	0	1	1	0	1	1	7
Saposnik et al. 2010 [60]	Y	1	0	1	0	0	1	0	0	1	1	5
Saposnik et al. 2016 [61]	Y	1	0	1	0	0	1	1	1	1	1	7
Shin et al. 2013 [62]	Y	1	0	1	0	0	1	1	0	1	0	5
Shin et al. 2014 [63]	Y	1	0	1	0	0	1	1	0	1	0	5
Shin et al. 2015 [64]	Y	1	0	1	0	0	1	1	0	1	1	6
Shin et al. 2016 [65]	Y	1	1	1	0	0	1	0	1	1	1	7
Sin et al. 2013 [66]	Y	1	0	1	0	0	1	1	0	1	1	6
Subramanian et al. 2013 [67]	N	1	1	1	0	0	1	1	0	1	1	7
Thielbar et al. 2020 [68]	Y	1	0	1	0	0	1	1	0	1	1	6
Tramontano et al. 2018 [69]	Y	1		1	0	0	1	1	0	1	1	6
Turolla et al. 2013 [70]	Y	1	0	1	0	0	1	0	0	1	1	5
Viana et al. 2014 [71]	Y	1	1	1	1	1	1	1	0	1	1	9
Yin et al. 2014 [72]	Y	1	0	1	0	0	1	1	0	1	1	6

**PEDro items: 1 Random allocation; 2 Concealed allocation; 3 Baseline Comparability; 4 Blind subjects; 5 Blind therapists; 6 Blind assessors; 7 Adequate follow-up; 8 Intention to treat analysis; 9 Between-group statistical comparisons; 10 Point estimates and variability

RevMan 5.4 was used to analyze the publication bias in this study. The funnel plots of Additional file 1: Fig. S1–S4 illustrate the evaluated weighted effect size, that is, the mean difference vs the standard error. The absence of publication bias is determined in the FMA-UE, BBT, WMFT and FIM outcomes by the symmetrical distribution of studies on the combined effect size.

#### FMA-UE meta-analysis

**Fig. 3:**
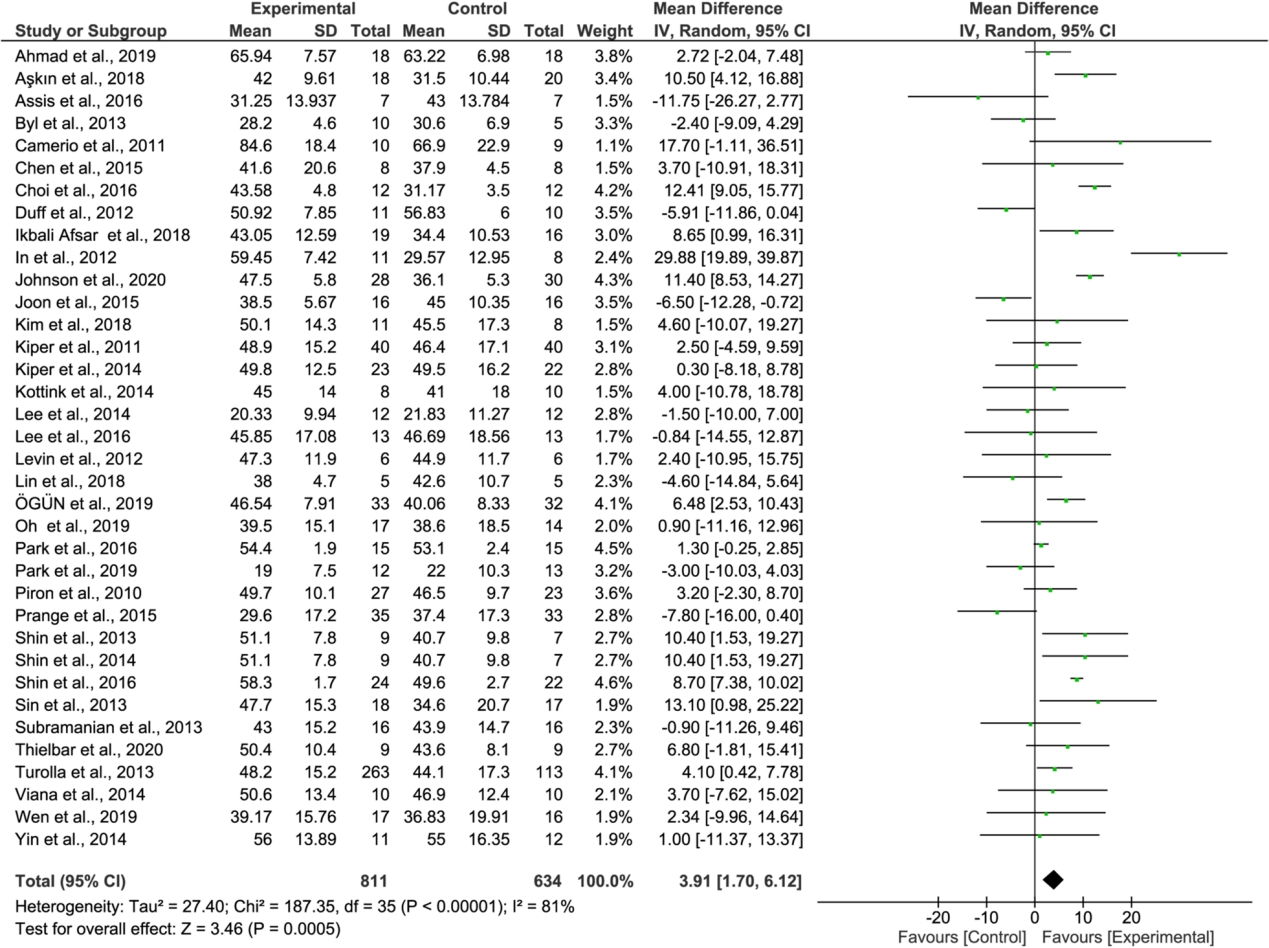
Forest Plot of the FMA-UE outcome

When analyzing the overall FMA-UE results, the recovery rate of the experimental group was significantly higher than that of the control group (36 studies, MD = 3.91, 95% CI = 1.70–6.12, P = 0.0005, Fig. 3). The heterogeneity is high (I^2^ = 81%).

#### BBT meta-analysis

**Fig. 4:**
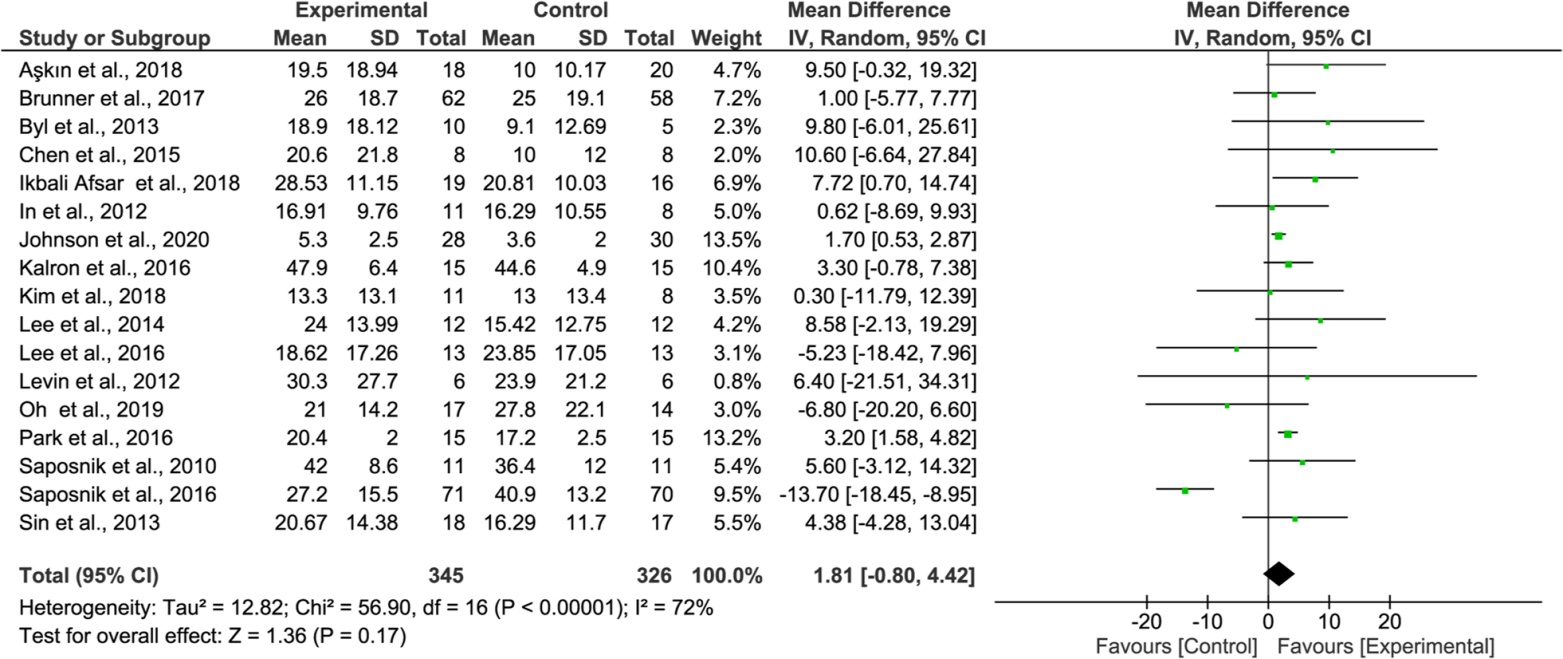
Forest Plot of the BBT outcome

For the overall BBT results, the total hand agility improvement of the experimental group was slightly higher than that of the control group but was not statistically significant (17 studies, MD = 1.81, 95% CI = − 0.80–4.74, P = 0.17, Fig. 4). The heterogeneity was quite high (I^2^ = 72%).

#### WMFT meta-analysis

**Fig. 5:**
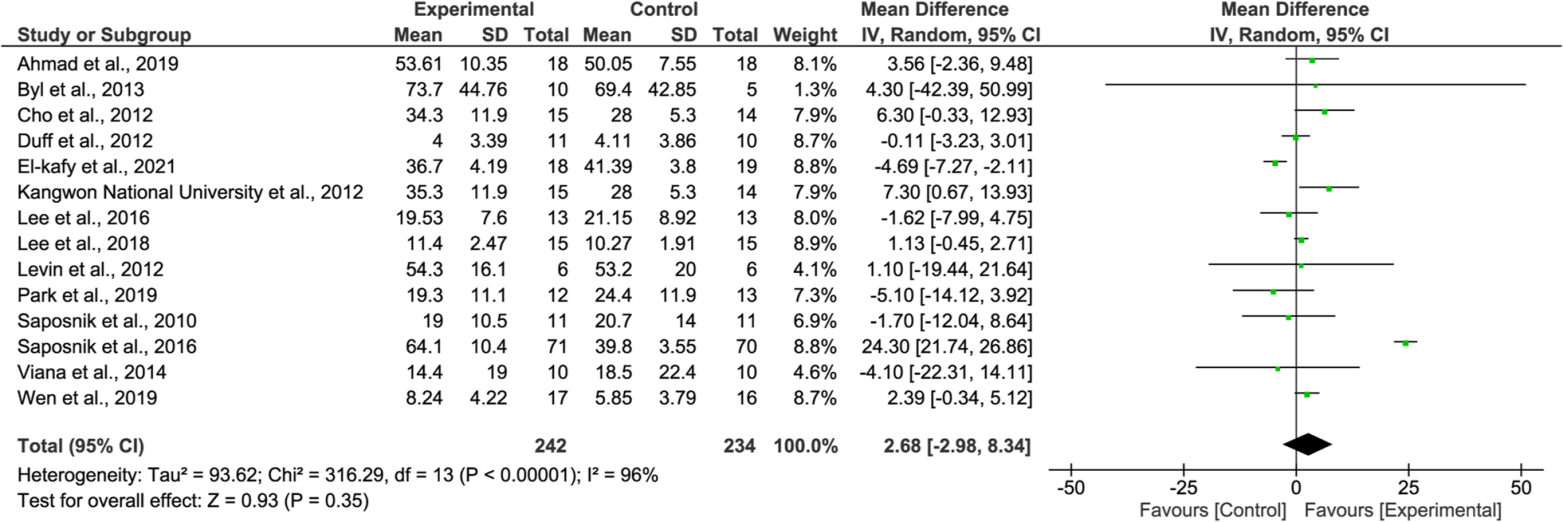
Forest Plot of the WMFT outcome

According to the overall WMFT results, the upper limb functionality of the experimental group was slightly higher than that of the control group, but was not statistically significant (15 studies, MD = 2.59, 95% CI = − 1.71–6.90, P = 0.24, Fig. 5). The heterogeneity is extremely high (I^2^ = 96%).

#### FIM meta-analysis

**Fig. 6:**
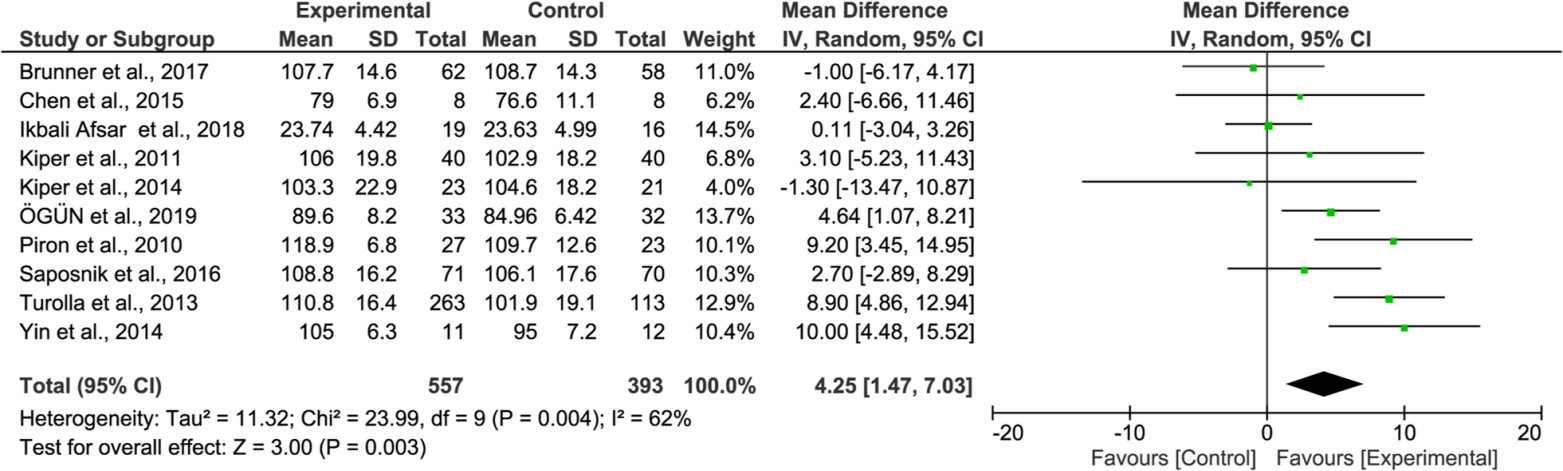
Forest Plot of the FIM outcome

For the overall FIM results, the improvement of the experimental group’s functional independence was higher than that of the control group and was statistically significant (10 studies, MD = 4.25, 95% CI = 1.47–7.03, P = 0.003, Fig. 6). The heterogeneity was slightly high (I^2^ = 62%).

#### Subgroup analysis

Regarding the high heterogeneity, subgroup analysis was performed by subdividing the studies based on the recovery stage after stroke: Chronic (more than 6 months), subacute (2 weeks to 6 months), and acute (about 2 weeks after onset), and results are shown below.

**Fig. 7:**
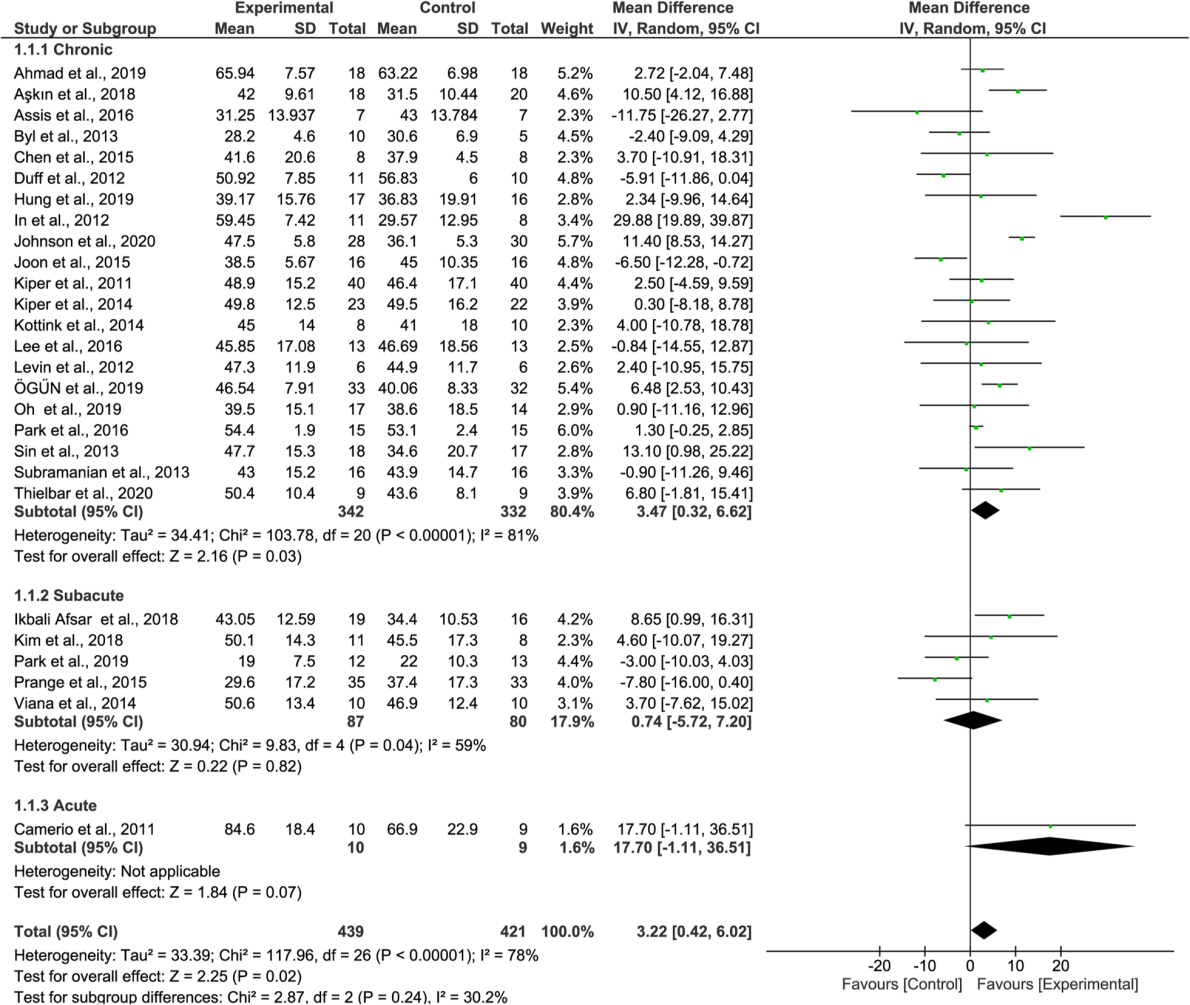
Forest Plot of the FMA-UE outcome regarding recovery stage

Regarding the FMA-UE results, the recovery rate of the patients at the chronic stage was significantly higher than in others (21 studies, MD = 3.47, P = 0.03, Fig. 7 upper panel).

**Fig. 8:**
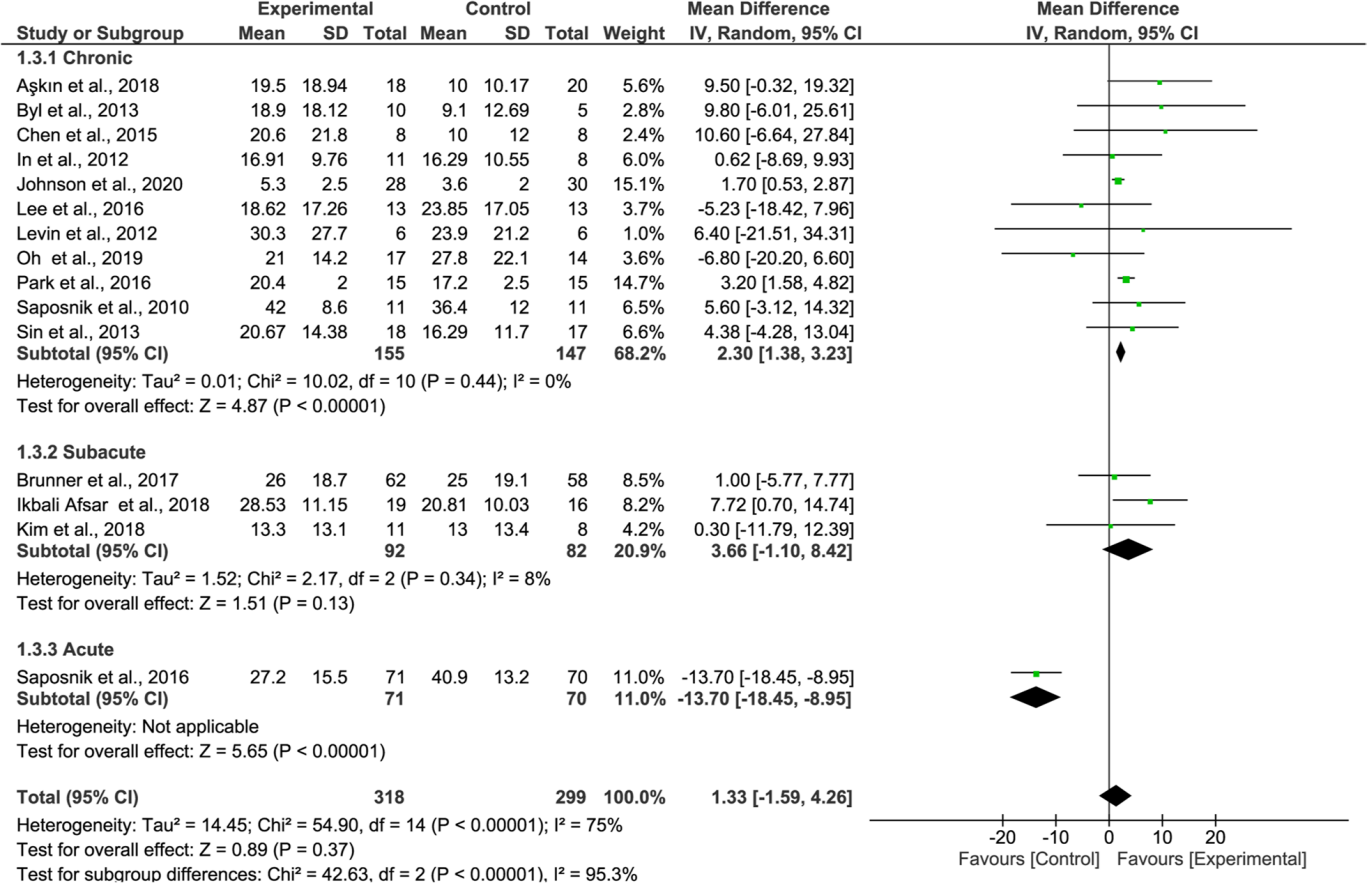
Forest Plot of BBT outcome regarding recovery stage

Regarding the BBT results, the recovery rate of the patients at the chronic stage was higher than in others (11 studies, MD = 2.330, P < 0.00001, Fig. 8 upper panel), with no heterogeneity.

**Fig. 9:**
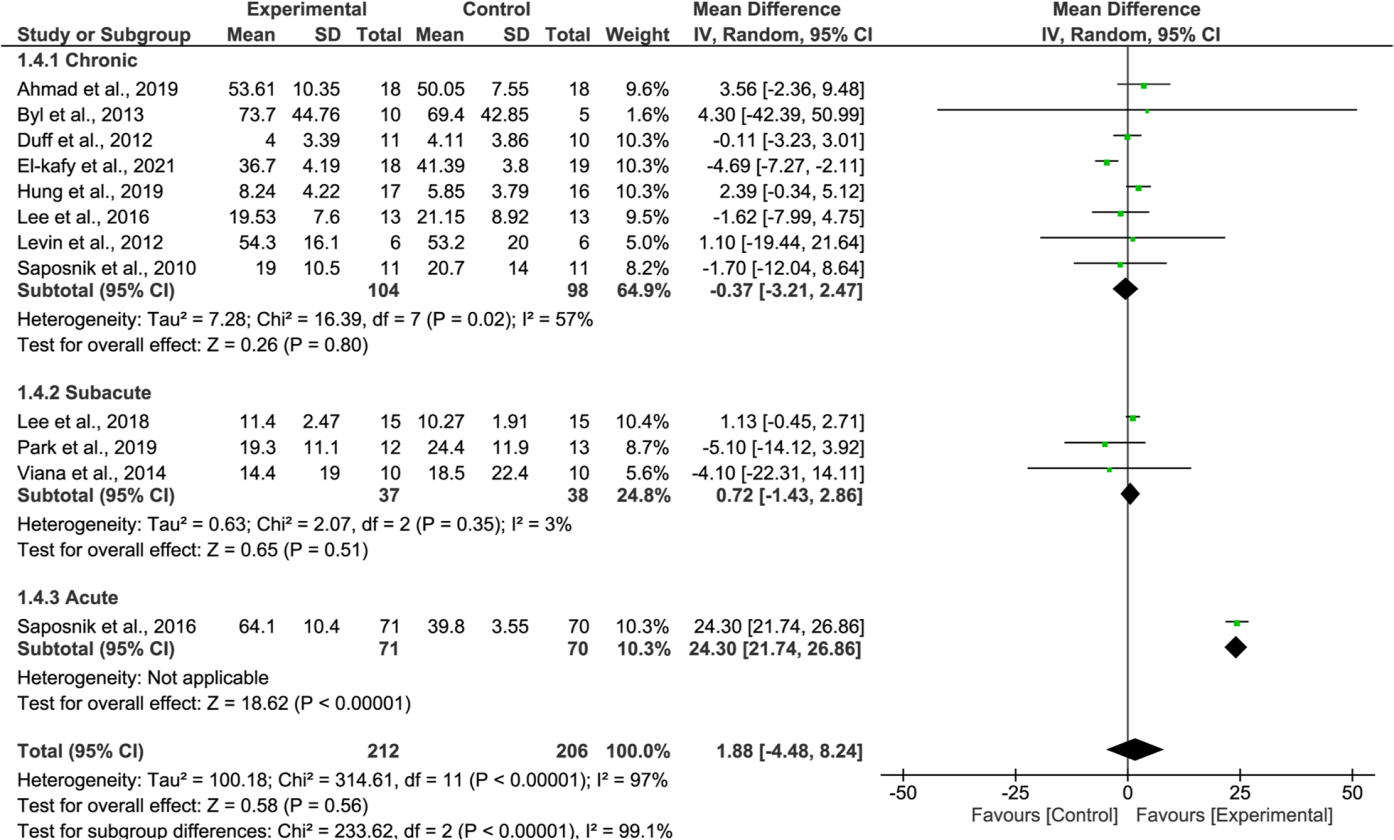
Forest Plot of the WMFT outcome regarding recovery stage

For the WMFT, the subgroup meta-analysis showed no significant effects neither patients with chronic stroke (8 studies, MD = − 0.37, P = 0.80, Fig. 9 upper panel) nor patients with subacute stroke (3 studies, MD = 0.72, P = 0.35, Fig. 9 middle panel).

**Fig. 10:**
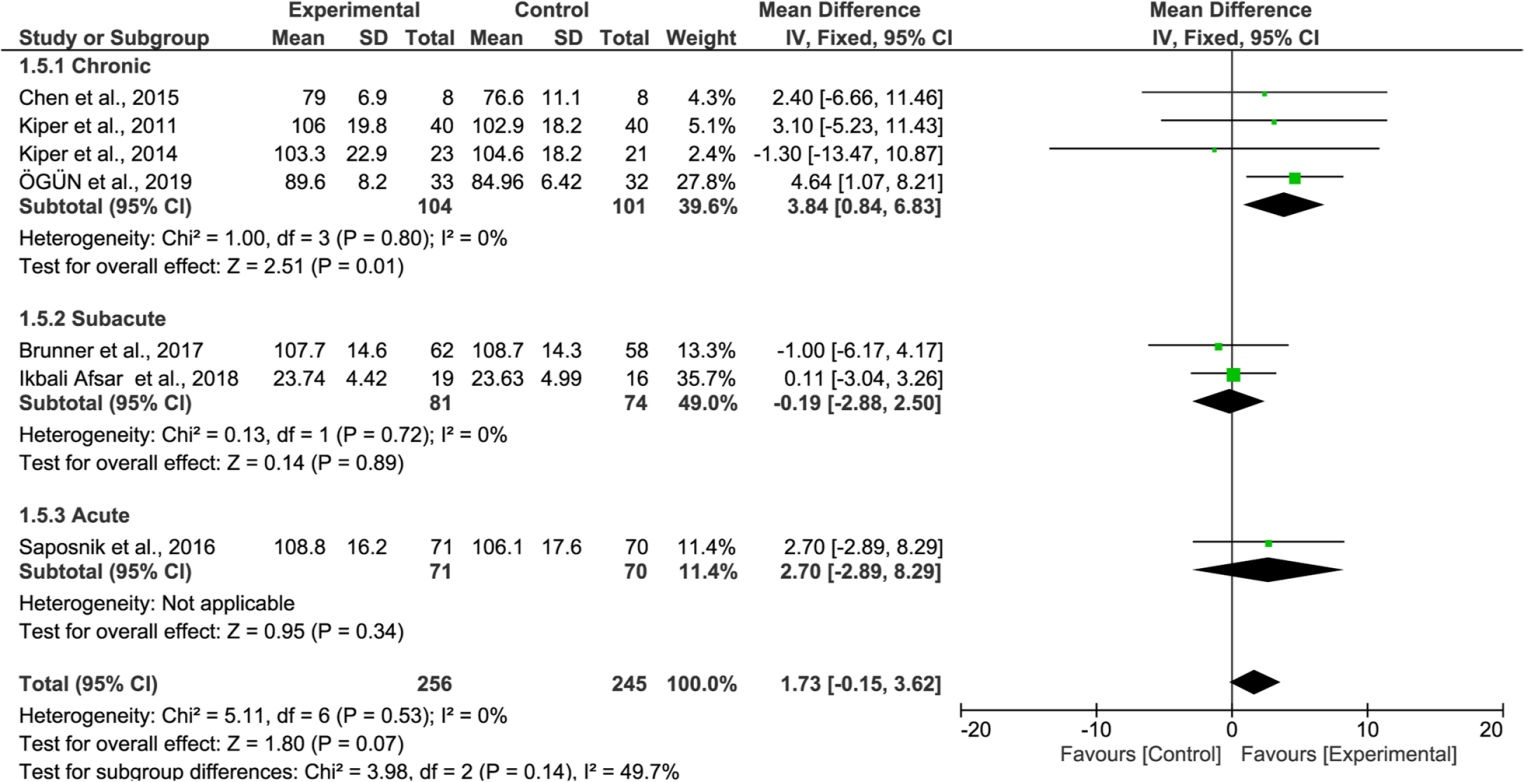
Forest Plot of the FIM outcome regarding recovery stage

In the subgroup meta-analysis of the FIM showed significant effects in patients with chronic stroke (4 studies, MD = 3.84, P = 0.01, Fig. 10 upper panel) while patients with subacute stroke did not (2 studies, MD = − 0.19, P = 0.89, Fig. 10 middle panel).

## Discussion

This review examines the VAMR-based treatment methods, UL function, and ADL in stroke outcome measures, and the effectiveness of VR-based therapies after a stroke compared with conventional therapies. A total of 4269 trials were screened and 50 RCTs of high reliability were established, involving 2271 participants who met the requirements for inclusion.

There are two main forms of VAMR training: commercially accessible games and customized systems specialized for upper extremity rehabilitation. With a customized system, it provides a great deal of rehabilitation flexibility for stroke patients based on their recovery progress [48]. Commercial games, it is simple to use and easy to obtain.

This review demonstrated that VAMR-based treatment showed positive effects to improve UL impairment and performance in ADL. According to the evaluation of the FIM results, VR has a significant impact on improving physical, mental and social functions, while the activity level of the arm also has improved. In particular, the subgroup analysis on the recovery stage reveals that patients with chronic stroke significantly improved better than those with subacute after VAMR training, with a lower heterogeneity.

The meta-analysis indicated that VAMR-based treatment improved in the FMA-UE and FIM, which are related to UL impairments and everyday functions, but not BBT and WMFT for the UL function assessments. According to Shin et al. [69], VR-based rehabilitation led to better functional gains in the distal upper extremity than conventional rehabilitation. As a scientifically proven intervention strategy for stroke patients, VR training may augment high-intensity, task-oriented treatment. The VR task-oriented treatment provided by Shin et al. [67] is challenging enough for the participation of patients. According to Flow Theory, enjoyment occurs when the task’s challenges and the participant’s skills are balanced, and for some patients, this balance was not reached in a certain portion of or until the end of the intervention period, depending on their stage of recovery and other factors [76]. Thus, VR content with varying degrees of difficulty to fit a diverse group of patients with varying degrees of stroke severity and at varying stages of recovery may improve participants’ self-perceived efficacy and positive attitude toward training. According to Yoshida et al.’s research [84], including adequate exercise content may boost motivation in stroke patients. VAMR therapy could energize stroke patients, preventing demotivation from standard therapy.

Additionally, the experimental group outperformed the control group in terms of UL improvement, as VR-based rehabilitation provided tailored feedback [76]. With this customized visual and audible feedback, VR group participants can improve incorrect postures continuously. According to Prochnow et al. [85] and Zhang et al. [86], this VR-based rehabilitative processing is a characteristic of the human mirror neuron system. Patients can benefit from action observation as well as mirror visual feedback provided by the VR technology in the form of augmented feedback which might facilitate the recovery of the UL function [86].

Furthermore, Turolla et al. [29] indicated that the post-treatment FIM scores were marginally higher in the VR sample than in the standard therapy population. Activities of daily living include a wide variety of instrumental ADL tasks such as shopping, mailing, paying bills, using of automatic teller machine, collecting trash, playing games, reading the news, preparing meals, etc. [87]. Numerous ADL tasks could be incorporated into VR devices, allowing for a variety of tasks to be completed throughout the therapy period, which is one of the primary variables affecting patients’ motivation. This may also explain why VR systems perform better than traditional treatments at improving daily functions.

There is no significant difference in the laboratory tests (BBT and WMFT) compared with conventional treatment, and Lee et al. [76] also reported that the VR group participants’ hand efficiency and dexterity were not superior to the conventional group participants for a variety of reasons, including the difficulties of optimizing hand function in patients with chronic stroke and the shortcomings of current VR technology in identifying minor gestures, such as those of the fingertips. This could be recognized as a limitation of VR technology, and these should be more focused on accuracy in future development.

Our findings are consistent with the results of various latest meta-analyses that found that the VR-based treatment reported more changes in the FMA-UE result relative to their controls. For example, referring to Mekbib et al.’s analysis [77], there was a significant improvement in upper limb function in the VR group, compared to the control group, in line with our results. However, there are some differences compared with recent analyses, Mekbib et al. stated that there was significant impairment on the upper hand activity level while there was no apparent improvement regarding the same outcome in our findings [77]. In addition, Wiley et al. [22] concluded there were no differences in daily function tests in the VR groups compared to the control groups, while our review concluded that there was a positive effect on ADL recovery. The differences might be due to the variety of trials included, as more studies were included in our review and the number of participants was more diverse.

### Limitations and recommendations for further research

This review has several limitations. First, one of the potential limitations is the diversity of VR treatment systems. We have not stratified the effectiveness of different treatments based on immersive and non-immersive VR, which might cause extraneous variability in the results. Another limitation is that our review includes studies that did not carry out subgroup analysis on different reality technologies. Furthermore, regarding the high heterogeneity of included studies, we have performed a subgroup analysis, however, there is still high heterogeneity in the analysis of each recovery stage according to the FMA-UE. Thus, mesh terms are not used in database searches, which means the search results may be limited. In addition, most findings of the outcome measures are related to motor functions, daily functions, and hand function measures, but not social functioning and cognition. This may be due to the inclusion criteria.

Future studies could be more focused on subgroup meta-analysis with stroke type and different reality technologies and include more high-quality trials examining the impact of VR, AR, and MR on hand functions. For further research on the outcome measures, not only randomized control trials but other studies could be included, for example, non-randomized controlled trials, cross-over studies, etc. With more studies examined, more types of assessment with high validity and reliability could be investigated. Besides, it could include lower limb or gait training studies in further research on the VAMR effectiveness.

## Conclusions

VAMR-based stroke rehabilitation has grown rapidly in recent years, and these therapies are regarded as beneficial and with significant advantages. For most stroke patients, full recovery of hemiplegic upper limb function is difficult; this significantly impairs their ADL and social interaction. Enhancing the functional use of the upper limbs following a stroke is important, as the majority of daily tasks require the use of the upper limbs. Our study examined the types of VAMR interventions used in stroke rehabilitation, identified the most commonly used outcome measures and evaluated the effect of VAMR interventions as compared to traditional therapy. To conclude, VAMR has a significant positive effect on improving the UL impairment (as measured using the FMA-UE) and daily functions (as measured using the FIM) but not for the UL function tests (as measured using the BBT and WMFT). Future studies should investigate the effects of VR, AR, and MR treatments compared with traditional treatment by subgroup analysis, for example, on the types of strokes.

## Supplementary Information

**Additional file 1.** **Table S1.** Search Strategy. **Figure S1.** Funnel plot of publication bias for FMA-UE outcomes. **Figure S2.** Funnel plot of publication bias for BBT outcomes. Figure S3. Funnel plot of publication bias for WMFT outcomes. **Figure S4.** Funnel plot of publication bias for FIM outcomes.

## Data Availability

All data generated or analyzed during this study are included in this published article and its additional file.

## Abbreviations

VAMR: Virtual, augmented and mixed reality
VR: Virtual reality
AR: Augmented reality
MR: Mixed reality
ADL: Activities of daily living
FMA-UE: Fugl-Meyer assessment for upper extremity
WMFT: Wolf motor function test
BBT: Box and block test
FIM: Functional independence measure
