# Supplementary material for: Examining the effectiveness of virtual, augmented, and mixed reality (VAMR) therapy for upper limb recovery and activities of daily living in stroke patients: a systematic review and meta-analysis
Source: J Neuroeng Rehabil. 2022 Aug 24;19:93. doi: 10.1186/s12984-022-01071-x (PMC9404551; doi:10.1186/s12984-022-01071-x)
Supplement: Supplementary file 1 — Additional file 1. Table S1. Search Strategy. Figure S1. Funnel plot of publication bias for FMA-UE outcomes. Figure S2. Funnel plot of publication bias for BBT outcomes. Figure S3. Funnel plot of publication bias for WMFT outcomes. Figure S4. Funnel plot of publication bias for FIM outcomes. [file 12984_2022_1071_MOESM1_ESM.pdf]

Additional file

Table S1. Search Strategy

Database	Keywords
ScienceDirect, PubMed, Web of Science and IEEE Xplore	(“virtual reality” OR “augmented reality” OR “mixed reality”) AND (“upper limb” OR “upper extremity” OR “arm” OR “hand”) AND (“stroke” OR “hemiplegia” OR “CVA” OR “cerebrovascular accident”)

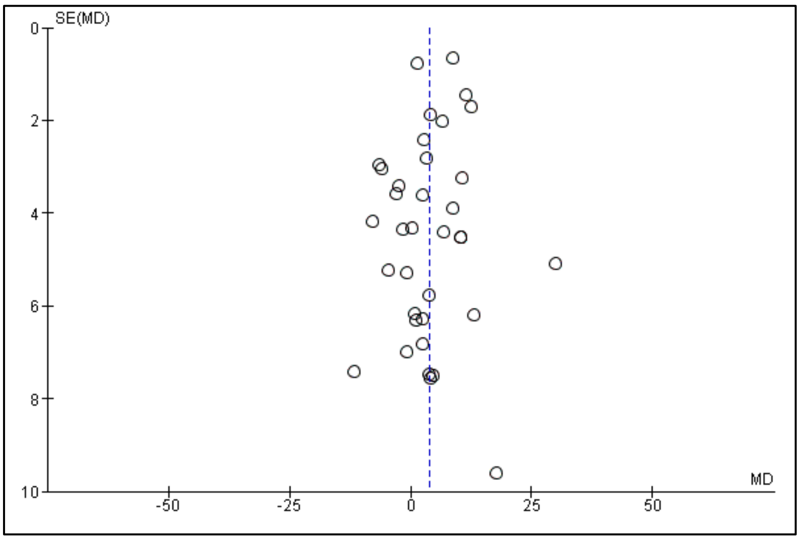

Figure S1. Funnel plot of publication bias for FMA-UE outcomes

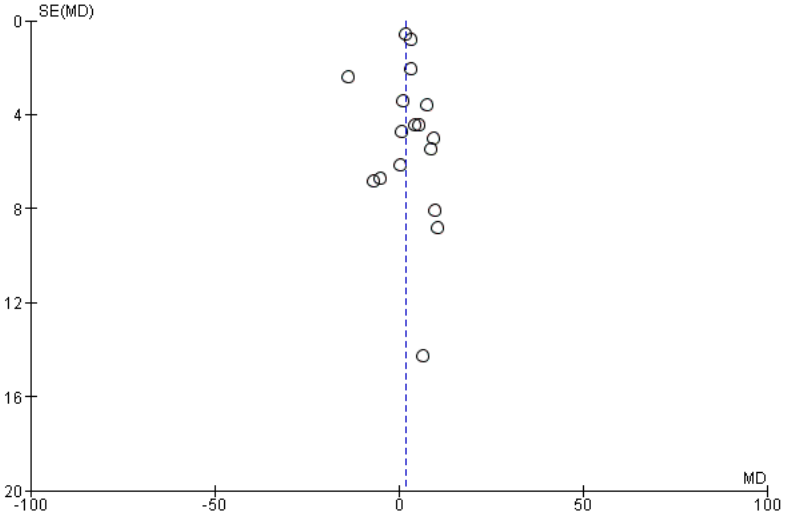

Figure S2. Funnel plot of publication bias for BBT outcomes

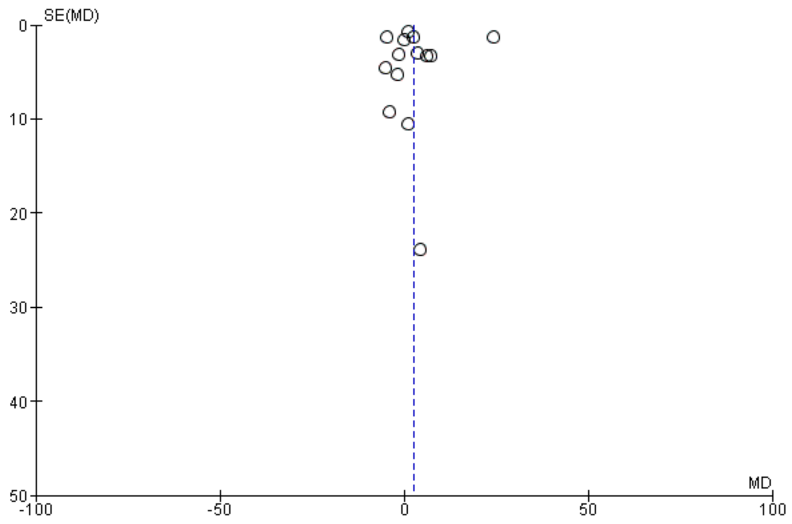

Figure S3. Funnel plot of publication bias for WMFT outcomes

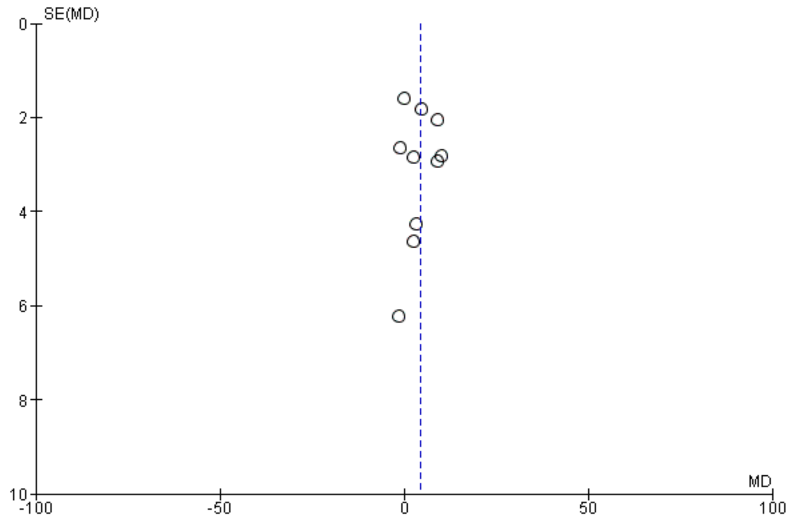

Figure S4. Funnel plot of publication bias for FIM outcomes
